# Lignin separation modification and high-value utilization based on low eutectic solvents: a review

**DOI:** 10.1186/s40643-026-01144-y

**Published:** 2026-10-20

**Authors:** Ruihan Sun, Chenchen Li, Ruike Liang, Yong Zhang, Rui Xu

**Affiliations:** 1 School of Energy and Environment Science, Yunnan Normal University, Kunming, 650500 China; 2 Key Laboratory of Intelligent Perception for Farmland Environment of Department of Education of Yunnan Province, Yunnan Normal University, Kunming, 650500 China; 3 Yunnan Provincial Field Scientific Observation and Research Station for Karst Agro-Ecological Environment and Low-Carbon Resource Utilization in Luoping, Qujing, 655800 China

**Keywords:** Deep eutectic solvents, Lignin extraction, Lignin structure, High-value utilization

## Abstract

**Abstract:**

Agricultural and forestry waste is rich in renewable lignin, which holds natural potential for the production of fine chemicals and functional materials. However, lignin is tightly cross-linked with cellulose and hemicellulose. Traditional extraction processes using strong acids and bases can easily cause lignin molecular condensation and damage to the molecular framework. At the same time, there are problems such as high energy consumption and heavy environmental burden, which seriously restrict its resource utilization. Due to their advantages of simple synthesis, high tunability, and environmental friendliness, deep eutectic solvents (DES) have become a research hotspot for the green and efficient extraction of lignin. This paper systematically reviews the latest research findings on DES-based extraction of lignin from agricultural and forestry waste, summarizes the characteristics of different types of DES components, investigates the effects of process parameters on lignin extraction efficiency and structural retention, and provides an in-depth analysis of the lignin's characteristic bonds, functional group composition, and other microstructural features. The study confirms that DES can achieve selective dissolution of lignin and effectively cleave ether bonds such as β-O-4; the resulting lignin has a low molecular weight, a narrow distribution, and a high content of phenolic hydroxyl groups, laying an excellent structural foundation for subsequent chemical modification and deep processing. The paper also further summarizes the high-value application potential of this type of lignin in fields such as the preparation of phenolic compounds and the synthesis of functional materials. At the same time, it objectively analyzes the practical challenges currently faced by DES technology in process optimization, solvent recycling, and industrial-scale upscaling, and makes targeted predictions regarding future key development directions. The aim is to provide a theoretical basis and practical reference for the iteration of green lignin separation technologies and the construction of high-value conversion systems for waste biomass.

**Graphical abstract:**

**Figure:**
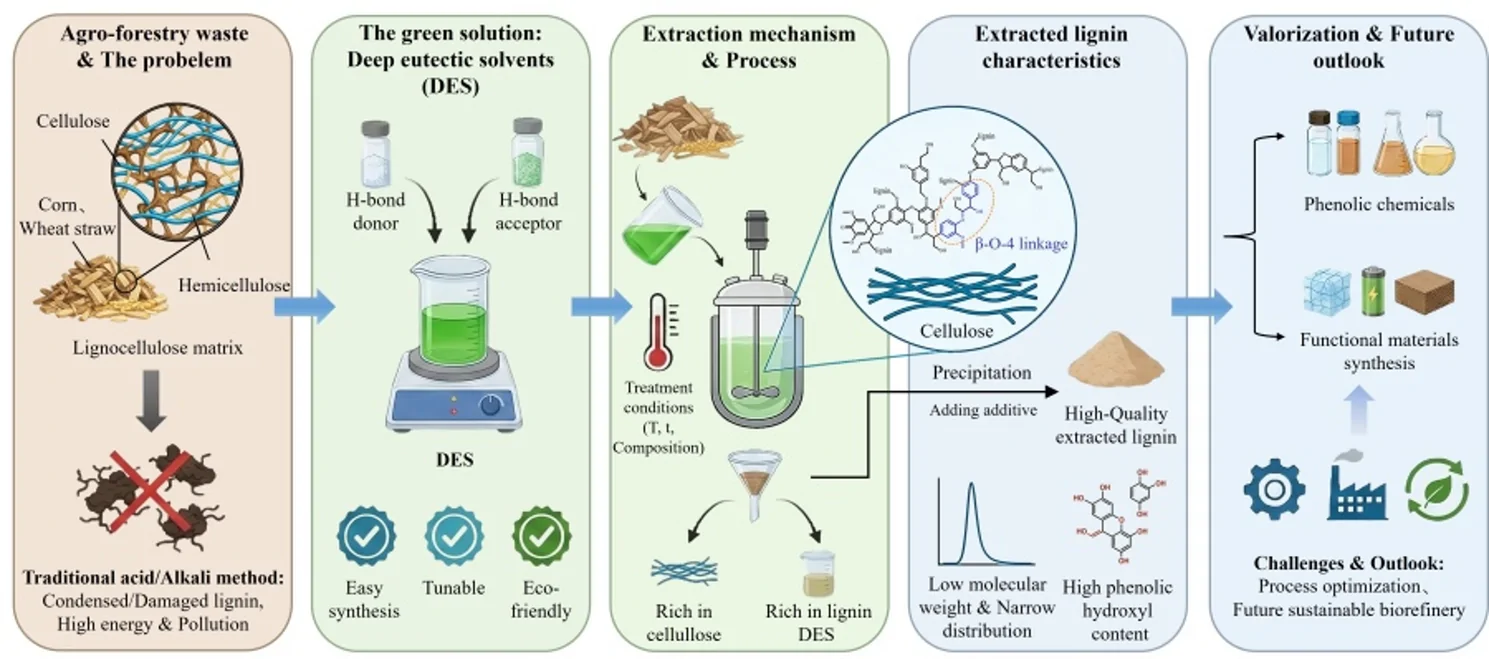

## Introduction

With the increasing depletion of fossil resources and the intensification of greenhouse gas emissions and environmental pollution, identifying green and sustainable pathways for biomass utilization has become a key focus in sustainable development research. Agricultural and forestry residues (including crop straw, forestry residues, wood processing waste) constitute a vital component of biomass resources, offering significant potential to reduce dependence on fossil resources, lessen environmental burdens, and advance the bioeconomy (Yıldız et al. 2024). Statistics indicate that globally, 18.15 billion tons of lignocellulosic biomass are generated annually, yet only 8.2 billion tons are utilized—a utilization rate far below its potential scale (Singh et al. 2022). Consequently, the high-value utilization of lignocellulosic biomass resources has emerged as a research hotspot.

As an abundant renewable resource, lignocellulosic biomass primarily consists of cellulose (30–50%), hemicellulose (20–30%), and lignin (15–30%) (Wang et al. 2020b). Among these, lignin is the most abundant aromatic polymer in nature and the second most prevalent natural organic polymer after cellulose (Mao et al. 2023; Xu et al. 2020). Lignin is composed of three structural units—p-hydroxyphenyl (H), guaiacyl (G), and syringyl (S)—linked by ether and carbon–carbon bonds, forming a highly amorphous, three-dimensional cross-linked network structure (Boerjan et al. 2003). Lignin's unique aromatic structure and abundant functional groups make it an ideal renewable feedstock for replacing fossil-based phenolic compounds, composites, and high-value-added chemicals (Jain et al. 2025).

However, practical applications face significant challenges in separating and extracting lignin. On one hand, within lignocellulosic biomass, lignin is tightly bound to hemicellulose and cellulose through complex covalent bonds and hydrogen bonds, forming a robust composite structure that severely hinders its dissolution and separation (Ralph et al. 2004). On the other hand, traditional separation methods like the Kraft process, though widely used industrially, typically require high-temperature, high-pressure, and strong acid conditions. These processes often involve substantial chemical usage and environmental pollution risks. During this treatment, lignin is highly susceptible to condensation reactions and structural failure, resulting in reduced activity and limited applicability of the separated lignin (Lu et al. 2025; Xie et al. 2024). Faced with the limitations of traditional separation methods, researchers are increasingly turning to novel solvents that are greener, milder, and more controllable. Ionic liquids (ILs) have garnered significant attention due to their biocompatibility and excellent thermal stability. However, their widespread industrial adoption remains constrained by high costs and difficulties in recovery, hindering large-scale implementation (Ji and Lv 2020).

In contrast, deep eutectic solvents (DES), as a novel green solvent, have demonstrated significant potential in the biorefining field since their initial report by Abbott et al. (2003). DES is a deep eutectic solvent (also known as a deep eutectic mixture) formed by mixing hydrogen bond acceptors (HBA) and hydrogen bond donors (HBD) in a specific molar ratio through hydrogen bonding interactions (Hou et al. 2018). Their unique physicochemical properties confer multiple significant advantages in lignin extraction: It can achieve highly selective dissolution of lignin, thereby efficiently separating cellulose, hemicellulose, and lignin (Abbott et al. 2001); compared to traditional strong acid or strong base treatments, DES processing is milder, reducing lignin condensation, repolymerization, and structural damage during extraction (Lobato-Rodríguez et al. 2023); low production costs, a simple process, and greater potential for recycling and reuse compared to other treatment methods. And most significantly, it preserves the native structural units of extracted lignin, facilitating subsequent high-value utilization (Chen et al. 2020b). Thus, DES-based lignin extraction technology provides a novel pathway for the high-value utilization of agroforestry residues.

In recent years, scholars both domestically and internationally have conducted extensive research on the extraction of lignin using DES and published many review articles. These studies have primarily focused on the effects of different types of DES on lignin extraction efficiency, the mechanisms of interaction with lignin, and the optimization of extraction processes. However, they have failed to systematically elucidate the intrinsic relationship among DES composition, processing methods, extraction efficiency, lignin structural characteristics, high-value applications, and there is a lack of in-depth comparative analysis regarding the differential effects of various DES systems and process conditions on lignin structure and physicochemical properties, making it difficult to guide targeted DES design. Against this backdrop, this review systematically summarizes recent research advances in extracting lignin from agricultural and forestry waste using DES processing methods. A systematic analysis is conducted across five aspects: DES composition, the relationship between DES process parameters and extraction efficiency, changes in lignin structural characteristics, alterations in lignin physicochemical properties, and the impact of DES processing on high-value applications of lignin. It comprehensively analyzes the high-value potential of DES-treated lignin, summarizes current research challenges, and outlines future development prospects. These findings aim to provide a comprehensive perspective on the efficient utilization of lignin from agroforestry residues, offering references and insights for subsequent researchers in DES system design, process development, and application promotion. At the same time, this is of significant importance for advancing the high-value utilization of agroforestry biomass and exploring green separation technologies for lignin.

## Composition of DES

DES is a blend of HBA and HBD. Due to strong hydrogen bonding between the components, the melting point of the mixture is significantly reduced, falling below that of either pure component, and typically exists as a liquid at room temperature (Lane et al. 2025). When extracting lignin, factors such as dissolution capacity, viscosity, pH, and chemical stability must be comprehensively considered to select appropriate categories of HBA and HBD. This enables the regulation of solvent physicochemical properties, thereby enhancing extraction efficiency and performance.

### Categories of HBA and HBD

The function of HBAs in DES is to act as hydrogen bond acceptors, forming stable hydrogen bonds with HBD molecules. Common HBAs are predominantly quaternary ammonium compounds (Gurkan et al. 2020). Among these, choline chloride (ChCl) is currently the most widely used HBA due to its low cost, good biodegradability, and relatively low toxicity. It can be combined with various HBDs such as urea, glycerol, and carboxylic acids to form a series of DES systems with distinct properties, making it extensively applied in lignocellulosic biomass fractionation studies (Perna et al. 2020; Rajapakse et al. 2025). Apart from ChCl, other quaternary ammonium salts, such as cetyltrimethylammonium bromide (CTAB) (Tang et al. 2023), tetrabutylammonium bromide (TBAB) (Zheng et al. 2024), and benzyltriethylammonium chloride (BTEAC) (Ge et al. 2024), can also serve as effective HBAs. They enhance delignification efficiency by disrupting the physical and chemical barriers formed by the dense cell wall structure, lignin–carbohydrate complexes (LCCs), and strong inter-component interactions in lignocellulosic biomass. The anion type of these quaternary ammonium salts influences hydrogen bond strength, mixture melting points, and solubility, necessitating rational selection based on target component characteristics. To further regulate DES properties, researchers have designed novel HBAs by synthesizing modified choline salts. For instance, altering alkyl chain length can significantly modulate the hydrophilic/hydrophobic balance of the solvent while maintaining strong hydrogen-bonding capacity. Such approaches enable the construction of more hydrophobic or dissolution-selective DES systems for selectively extracting plant components with different polarities (Mastellone et al. 2023). Additionally, certain natural or bio-based molecules demonstrate potential as HBAs. Betaine, with its high renewability and low toxicity, serves as an environmentally friendly HBA (Fan et al. 2024; Hasanah et al. 2025). Certain amino acids, such as glycine and lysine, can also serve as HBAs. They not only mildly dissolve lignin but also allow for partial chemical modification, offering additional possibilities for designing functionalized DES or natural azeotropic solvents (NADES) (Li et al. 2025b).

HBD, as another key component constituting DES, primarily functions by forming hydrogen bond networks with HBA to lower the mixture's melting point and regulate the physicochemical properties of DES, such as melting point, viscosity, and density (Yang et al. 2024). Organic acids are a significant class of HBDs, such as lactic acid (LA) and formic acid (FA). Their strong hydrogen-bonding capabilities not only adjust the acidity of the solvent but also effectively disrupt the ester bonds and hydrogen-bond networks within lignin (Hong et al. 2020; Li et al. 2023b). Consequently, these acidic DES often exhibit outstanding delignification capabilities. Studies indicate that compared to glycerol and urea, LA possesses solubility parameters more compatible with lignin dissolution (Jessop et al. 2012; Yu et al. 2011). For instance, DES synthesized from ChCl and LA, characterized by higher acidity and lower viscosity, effectively decomposes lignin-polysaccharide complexes and promotes lignin dissolution (Thi and Lee 2019). Polyalcohols are another common class of HBDs, represented by glycerol (Gly) and ethylene glycol (EG). Gly contains three hydroxyl groups, while EG contains two, enabling them to interact with HBAs through multiple hydrogen bonds and form stable DES networks. When combined with ChCl, such DES exhibit high polarity and excellent solubility for biomacromolecules like lignin (Cheng et al. 2024). Urea is also one of the most frequently used HBDs in DES systems. The typical ChCl/urea system (usually at a 1:2 molar ratio) not only exhibits significantly reduced melting points but also demonstrates good dissolution capabilities in many biomass pretreatments (Agrawal et al. 2025). Beyond these common categories, polycarboxylic acids like citric acid (CA) also frequently serve as HBDs. Their multiple carboxyl groups enable formation of more complex hydrogen-bond networks with HBAs, enhancing DES stability while significantly modulating system polarity, acidity, and solubility properties (Jin et al. 2025; Savi et al. 2019). Furthermore, some studies also indicate that other dicarboxylic acids and tricarboxylic acids, such as malic acid and tartaric acid, can also participate in DES construction as HBDs. These HBDs also demonstrate unique advantages in influencing solvent polarity and dissolving biomass (Fernandes et al. 2024; Qin et al. 2023; Wu et al. 2025a).

### Composition and characteristics of the binary DES

In the DES system, a DES composed of one HBA and one HBD mixed at a specific molar ratio, known as a binary DES, represents the most fundamental and extensively studied type of eutectic solvent. Different combinations of HBA and HBD determine the physicochemical properties of the system, such as melting point, viscosity, density, polarity, and solubility, which in turn influence its application performance (Smith et al. 2014) (see Table 1). Among numerous binary DES, systems based on ChCl are particularly common and can be combined with other types of HBDs to form binary DES with diverse functionalities.

**Table 1:** Physical and chemical properties of binary DESs

DES	Molar ratio	Melting point (°C)	Viscosity (Pa s)	Density (g cm^−3^)	References
ChCl: LA	1:10	18.34	0.87	1.2022	Liu et al. (2020a)
ChCl: Citric acid monohydrate	3:1	159.55	7.27	2.64	Shafie et al. (2019)
TBAB: Octanoic Acid	1:4	286.5	15.67	0.9322	Zheng et al. (2024)
ChCl: Diethanolamine	1:6	292.22	6.3	1.0616	Murshid et al. (2019)

ChCl: choline chloride; LA: lactic acid; TBAB: tetrabutylammonium bromide

For example, combination with polyols or amides can yield DES with distinct properties. ChCl-urea forms a liquid with a melting point of approximately 12 °C at a 1:2 molar ratio, significantly lower than the melting points of choline chloride (302 °C) and urea (133 °C) (Abbott et al. 2003); ChCl-EG at a 1:2 molar ratio possesses low viscosity (≈ 36 cP), reasonable density (≈ 1.12 g cm^−3^), improved flowability, and high ionic conductivity, making it suitable as an electrolyte or extractant(Smith et al. 2014); DES composed of ChCl-Gly can have its physical properties, which viscosity and density, can be tuned by varying the molar ratio, and is commonly used for extracting polar compounds from natural products (Sazali et al. 2023).

When combined with organic acids such as FA and LA, it was found that DES exhibits enhanced hydrogen-donating capacity and reduced hydrogen-accepting capacity, while the number of intermolecular hydrogen bonds increases and their average strength decreases, effectively improving the solubility of lignin (Li et al. 2021a; Oh et al. 2020). At the same time, ChCl-CA, ChCl-oxalic acid (OA), and ChCl-malonic acid also exhibit strong hydrogen bonding interactions, significantly lowering the system's melting point and imparting higher acidity to the solvent. These acidic DES demonstrate excellent performance in metal ion leaching and extraction separation, thus attracting attention in environmental analysis and material extraction fields (Almeida et al. 2020; Samarov et al. 2017; Shekaari et al. 2023). Other studies have employed natural DES composed of ChCl and sugar-based hydrogen bond donors like glucose and fructose, demonstrating excellent compatibility in biomacromolecule extraction and protein separation (Karabulut et al. 2025; Mqoni et al. 2025).

Beyond choline-based DES, research has also explored binary systems composed of other HBA structures. For instance, DES formed with TBAB as the HBA and Gly, EG, or 1,3-propanediol exhibit parameters such as density, viscosity, and conductivity that are modulated by the HBD type, molar ratio, and temperature, thereby influencing the solvent's fluidity and separation performance (Yusof et al. 2014). Furthermore, DES formed by methyltriphenylphosphonium bromide as the HBA with Gly, EG, and triethylene glycol have been employed for selectively removing Gly and its derivatives from biodiesel, demonstrating the application advantages of non-choline-based DES in specific separation fields (Shahbaz et al. 2011).

Overall, binary DES enable effective control over solvent physicochemical properties through the judicious selection of HBA and HBD types and their molar ratios.

### Composition and characteristics of the ternary DES

With increasing demands for the designability and functionalization of DES, research has expanded from simple binary systems to ternary systems. By introducing a third component, more precise control over physicochemical parameters such as solvent polarity, acidity, and viscosity has been achieved (Sathasivam et al. 2025) (see Table 2). Ternary DES not only modifies the density and structure of hydrogen-bonding networks but also significantly improves the solubility selectivity and mass transfer properties of the system. This enables more efficient applications in fields such as biomass pretreatment, material synthesis, and energy storage (Sathasivam et al. 2025).

**Table 2:** Physical and chemical properties of ternary DESs

DES	Molar ratio	Melting point (°C)	Viscosity (Pa s)	Density (g cm^−3^)	References
ChCl: Gly: Polyethylene glycol 600	1:2:4	18.4	0.1823	1.135	Singh et al. (2018)
ChCl: Urea: L-arginine	1:2:0.05	–	1.2337	1.2022	Chemat et al. (2016)
Tetraoctylammonium bromide: Tetrabutylammonium bromide: Methyl-(2R)-2-hydroxy-3-phenylpropanoate	1:1:4	–	0.748	1.05	Teague et al. (2025)
Tetraoctylammonium bromide: Tetrabutylammonium bromide: (R)-ethyl mandelate	1:1:4	–	0.6	1.05	Teague et al. (2025)

ChCl: choline chloride; Gly: glycerol

In biomass pretreatment, the incorporation of a third component into conventional binary DES systems centered on choline salts enables the construction of ternary DESs, which can further improve the pretreatment efficiency of lignocellulosic biomass. For example, a ternary DES formed by adding p-hydroxybenzoic acid to a ChCl-LA binary DES achieved a lignin extraction efficiency of 61.91%, representing an approximately 1.86-fold improvement over the original binary system( Ge et al. 2025). Similarly, introducing EG as an additional HBD into the alanine-LA system enables the formation of a ternary DES, which facilitates the selective separation of recalcitrant lignin from lignocellulosic biomass and thereby enhances lignin removal efficiency (Ee et al. 2023). Furthermore, studies have designed various ternary DES comprising ChCl and guanidine hydrochloride as HBA, EG, propylene glycol, and Gly as polyol-based HBD, and toluene sulfonic acid as an acidic HBD. These systems efficiently remove lignin and xylan at high solid-to-liquid ratios and lower temperatures while maintaining stable degradation efficiency (Chen et al. 2019b).

In material synthesis, ternary DES exhibit superior performance and selectivity in material preparation due to their unique solvent environment. For example, using a ternary DES of ChCl-urea-MnCl_2_ (1:1:0.25) as a precursor medium, Mn_3_O_4_@N-doped carbon materials can be directly synthesized via thermal treatment—a product difficult to achieve directly in binary DES systems (Zdolšek et al. 2022). In another study, ChCl-urea-Gly, owing to its uniform dispersion and favorable physicochemical properties, was employed to construct a lignin eutectic gel, which was further processed into porous carbon materials for supercapacitor electrodes (Ma et al. 2021).

In the field of energy storage, the incorporation of a third component can broaden the operating temperature range, enhance ion conductivity, and reduce viscosity, thereby optimizing electrochemical performance. When Gly is added to ChCl-EG to form a ternary DES as an electrolyte, it can increase the power density of the battery by 38.2% (Lu et al. 2022b). The ChCl-EG-urea (1:2:1) DES exhibits low viscosity (3.39 cP) and high conductivity (13.41 mS/cm), showing excellent electrochemical performance (Zhong et al. 2020). For CO_2_ capture, Kafash et al. (2025) found that the [C4DMAP]Br-boric acid-EG ternary DES reduces solvent viscosity and increases contact between reactive sites and CO_2_, thereby enhancing the selectivity of the epoxide-CO_2_ reaction. Sze et al (2014) demonstrated that the ChCl-urea-Gly (1:2:1) system exhibited a 20% higher CO_2_ solubility (0.25 mmol/g) at 298 K and 101.3 kPa compared to ChCl-urea (0.15 mmol/g), attributed to viscosity reduction (Ghazali et al. 2020). In addition, the ternary system composed of ChCl, monoethanolamine, and water exhibits higher CO_2_ adsorption efficiency than the binary system due to its superior physical properties (Abdrabou et al. 2023).

In summary, ternary DES outperform traditional binary systems in multiple leading-edge applications due to their stronger component functional synergies and higher designability. As research remains in its infancy, the types of ternary DES additives are currently limited to materials such as organic acids, polyols, and metal salts.

## Effect of process parameters on lignin extraction efficiency

The physicochemical properties of DESs determine their intrinsic lignin dissolution ability. However, process parameters, including extraction time, reaction temperature, solid-to-liquid ratio, and auxiliary techniques, are key factors controlling the lignin separation process. These parameters not only directly influence lignin extraction efficiency and purity but also regulate important structural characteristics of lignin, such as molecular weight distribution and functional group reactivity, thereby determining its potential for subsequent high-value-added applications. Therefore, achieving high lignin recovery requires a comprehensive evaluation of the effects of process parameters on both lignin structural preservation and the feasibility of industrial-scale application. Table 8 summarizes the effects of different DES extraction conditions on lignin recovery from agricultural and forestry residues.

### Time

During DES-based lignin extraction, extraction time determines the extent of solvent penetration into lignocellulosic biomass cell walls and the cleavage degree of LCC linkages. At the early stage of extraction, extending the treatment duration facilitates the diffusion of DES molecules into the biomass matrix, thereby promoting lignin depolymerization and dissolution. To further elucidate the influence of extraction time on lignin recovery, previous studies investigating different treatment durations were summarized (Table 3). For instance, during the ChCl-LA treatment of willow branches at 120 °C, lignin recovery increased linearly with an extension of treatment time from 6 to 12 h (Li et al. 2017). Moderate treatment conditions can effectively disrupt chemical linkages within LCC structures while preserving the native reactive functional groups of lignin. Consequently, the recovered lignin exhibits a relatively low molecular weight and narrow molecular weight distribution, providing a favorable structural basis for subsequent high-value applications, such as high-reactivity adhesives and antioxidants.

**Table 3:** Influence of extraction time on the efficiency of lignin extraction

Raw material	DES	Temperature (°C)	Time (h)	Lignin yield (%)	Effect	References
Willow branches	ChCl: LA	120	6	~ 67	Changes became marginal after 12 h, accompanied by the dissolution and degradation of carbohydrates	Li et al. (2017)
			12	91.80		
			42	94.20		
Date palm waste	ChCl: FA	120	2	~ 12	Yield decreases with extended time due to over-degradation	Dandash et al. (2025)
			4	22.85		
			6	~ 14		
Wheat straw	ChCl: LA	120	6	53.20	Yield increases with extended time	Ma et al. (2022)
			12	71.60		
		150	6	81.50	Yield decreases with extended time	
			12	47.80		
Salix babylonica	ChCl: OA	130	1	17.20		
			5	~ 53	Yield increases with extended time and plateaus after 7 h	Wu et al. (2025b)
			7	~ 63		
			9	66.10		

ChCl: choline chloride; LA: lactic acid; FA: formic acid; OA: oxalic acid

Nevertheless, extending the extraction time does not necessarily lead to improved lignin recovery. Excessive treatment durations may result in reduced extraction efficiency and severe degradation of lignin structures. Once the treatment time exceeds the optimal duration, the lignin depolymerization and dissolution effects of DESs gradually reach a plateau. Meanwhile, the dissolved lignin fragments become highly susceptible to secondary condensation reactions and excessive degradation under high-temperature DES conditions. Previous studies on DES pretreatment of poplar and date palm agricultural residues demonstrated that lignin recovery reached its maximum after 4 h of treatment, whereas further extension of the reaction time to 6–12 h significantly decreased both lignin yield and purity, accompanied by reduced molecular weight and deteriorated thermal stability of the recovered lignin (Dandash et al. 2025; Tian et al. 2023).

### Temperature

Among the operational parameters, temperature plays a pivotal role in regulating the physicochemical properties of DESs, as well as the mass transfer and lignin dissolution processes during extraction. At a fixed extraction time, moderate temperature elevation (typically 80–150 °C) can effectively reduce DES viscosity, enhance molecular mobility, and facilitate DES penetration into lignocellulosic matrices, thereby accelerating the cleavage of ether and ester linkages within LCCs (Okur and Koyuncu 2020). To further elucidate the influence of temperature on lignin extraction performance, representative studies under different temperature conditions were selected from the literature and summarized in Table 4. For example, during the ChCl-LA treatment of wheat straw, increasing the temperature from 90 to 150 °C within 6 h significantly improved lignin yield and purity, reaching 81.54% and 91.33%, respectively (Ma et al. 2022). Similarly, studies on willow pretreatment using ChCl-Gly, ChCl-urea, and ChCl-LA showed that the residual lignin content in the solid fraction decreased with increasing temperature, whereas lignin recovery increased progressively (Li et al. 2017). These results demonstrate that moderate heating facilitates interactions between DES molecules and lignin, thereby promoting efficient lignin dissolution.

**Table 4:** Effect of temperature on lignin extraction performance

Raw material	DES	Time (h)	Temperature (°C)	Lignin yield, purity	Effect	References
Wheat straw	ChCl: LA	6	90	9.3%, 62.8%	Increasing temperature promotes lignin release, enhancing both extraction yield and purity	Ma et al. (2022)
			120	53.2%, 86.5%		
			150	81.5%, 91.3%		
Willow	ChCl: LA	6	90	~ 9.5%, –	Elevated temperatures facilitate lignin dissolution while reducing solid residue recovery	Li et al. (2017)
			110	~ 34.5%, –		
			120	52.43%, –		
Acacia wood	ChCl: CA: LA	4	120	~ 52%, –	Temperatures > 160 °C yield a black foamy mixture, likely due to DES degradation or lignin condensation reactions	Fernandes et al. (2024)
			140	~ 65%, –		
Red pine	ChCl: LA	24	100	~ 17.9%, –	Reduction in functional group content and increased polydispersity of molecular weight distribution	Park et al. (2022)
			120	~ 21.7%, –		
			130	~ 29%, –		

ChCl: choline chloride; LA: lactic acid; CA: citric acid

Nevertheless, excessive reaction temperatures can induce undesirable chemical transformations and compromise the downstream valorization potential of lignin. When the temperature exceeds an appropriate range, intensive side reactions may occur, including thermal degradation of DES components and extensive condensation or cross-linking of dissolved lignin fragments (Fernandes et al. 2024). For instance, in an LA-CA-ChCl system, increasing the temperature from 120 to 140 °C significantly enhanced lignin extraction efficiency; however, further elevation to 160–180 °C resulted in the formation of a black foamy product and a decline in extraction performance (Fernandes et al. 2024). Moreover, elevated temperatures can accelerate the degradation of reactive functional groups in lignin. During ChCl-LA treatment of red pine, the contents of methoxy, phenolic hydroxyl, and aliphatic hydroxyl groups were lower at 130 °C than at 100 °C, while the polydispersity index (PDI) increased from 1.6 to 3.4 (Park et al. 2022). These findings indicate that excessive thermal treatment promotes uncontrolled secondary cross-linking among lignin molecules, resulting in broader molecular weight distributions and reduced molecular uniformity.

Overall, temperature exerts a dual influence on DES-based lignin extraction, and its optimization directly determines lignin structural characteristics and subsequent valorization pathways. Relatively mild temperatures (100–120 °C) may lead to slower extraction kinetics but enable better preservation of phenolic and aliphatic hydroxyl groups while suppressing condensation reactions. Consequently, lignin with high chemical reactivity and low polydispersity can be obtained, making it suitable as a precursor for high-reactivity resin adhesives, antioxidants, and polyurethane materials. In contrast, when lignin is targeted for thermally stable carbon materials or carbon fiber precursors, moderately elevated temperatures may be acceptable because controlled condensation reactions can enhance aromatic network formation and carbon skeleton stability (Kim et al. 2014). Therefore, in practical process design, the optimal extraction temperature should be carefully selected according to the desired lignin properties, while balancing extraction efficiency, structural preservation, and energy consumption.

### Solid–liquid ratio

The solid-to-liquid ratio plays a decisive role in governing mass transfer efficiency and the solvent's effective dissolution capacity during DES extraction, directly dictating the extent of contact between the solvent and the biomass substrate. At lower solvent loadings, the insufficient liquid volume fails to thoroughly wet and submerge the biomass particles. This imposes substantial mass transfer resistance, hindering adequate lignin dissolution and leading to generally low delignification efficiency. To elucidate the influence of the solid-to-liquid ratio on DES-mediated extraction, key outcomes across various solid-to-liquid ratios are summarized in Table 5.

**Table 5:** Effect of solid-to-liquid ratio on lignin extraction performance

Raw material	DES	Temperature, time	solid–liquid ratio	Lignin extraction performance	Effect	References
Wheat straw	ChCl: LA	110 °C, 6 h	1: 20	~ 45% lignin-removal rate	Increasing solvent volume promotes thorough biomass wetting and improves contact efficiency between DES and lignin. However, excess solvent provides limited contribution to further dissolution	Li et al. (2023c)
			1: 40	~ 83% lignin-removal rate		
			1: 60	~ 86% lignin-removal rate		
			1: 80	~ 90% lignin-removal rate		
Corncob	ChCl: Ethanolamine	50 °C, 12 h	1: 10	77.22% delignification efficiency	An increased solvent volume facilitates lignin dissolution. However, further additions yield diminishing returns once saturation is reached	Jing et al. (2022)
			1: 15	78.5% delignification efficiency		
			1: 20	79.2% delignification efficiency		
			1: 25	83.12% delignification efficiency		
			1: 30	82.99% delignification efficiency		
Sugarcane bagasse	ChCl: Urea	150 °C, 12 h	1: 10	68.88% lignin removal	Appropriately increasing the solvent ratio aids in lignin removal. If efficiency is prioritized, elevating temperature should be favored over excessively increasing the solvent ratio	Varilla-Mazaba et al. (2022)
			1: 20	72.53% lignin removal		
		120 °C, 12 h	1: 10	41.45% lignin removal		
			1: 20	46.38% lignin removal		

ChCl: choline chloride; LA: lactic acid

As the solvent proportion increases moderately, system viscosity decreases, significantly enhancing the driving force for mass transfer. This facilitates more thorough contact between the solvent and the cell wall matrix, driving a pronounced increase in lignin yield. For instance, in ChCl–LA extraction of wheat straw lignin, increasing the solid-to-liquid ratio from 1:20 to 1:40 substantially improved the lignin removal efficiency (Li et al. 2023c). Similarly, during ChCl–urea extraction of sugarcane bagasse, a 1:20 ratio exhibited superior lignin extraction performance compared to 1:10 (Varilla-Mazaba et al. 2022).

However, excessively increasing the solid-to-liquid ratio not only has a very limited effect on improving extraction efficiency but also leads to a series of negative consequences. Beyond a certain threshold, lignin dissolution approaches saturation, resulting in negligible yield gains (Jing et al. 2022). More critically, excessively high solvent loadings (> 1:50) tend to induce over-depolymerization of solubilized lignin fragments. This paradoxically reduces overall lignin recovery while compromising both its molecular weight distribution and structural stability (Liao et al. 2024). Furthermore, high solvent ratios demand dramatically larger volumes of DES, which inflates raw material costs and increases antisolvent consumption during subsequent precipitation. This significantly elevates the energy burden and equipment throughput required for downstream DES evaporation, concentration, and recycling.

Thus, the optimization of the solid-to-liquid ratio should balance lignin structural modification with the overall economic feasibility of industrial production. For fine chemical feedstocks requiring high purity and a specific degree of depolymerization, a moderately high solid-to-liquid ratio can be adopted to ensure sufficient mass transfer (Han et al. 2024). Conversely, for industrial applications targeting large-scale sustainable production, a lower solid-to-liquid ratio (e.g., 1:10) should be prioritized. This strategy minimizes solvent consumption and the associated energy requirements for solvent recovery while maintaining satisfactory extraction performance, thereby improving the overall advantages and practical feasibility of the DES pretreatment process (Xie et al. 2026).

### Auxiliary agents

Beyond optimizing physical process parameters such as temperature and reaction duration, introducing appropriate chemical additives into DES systems represents another highly effective strategy for boosting lignin extraction efficiency. These auxiliary agents are predominantly Lewis acids, which synergistically facilitate lignin isolation from biomass by modulating solvent polarity, hydrogen-bonding networks, and system acidity (He et al. 2024b). To further elucidate the influence of additives on DES-mediated lignin extraction, representative extraction outcomes are summarized in Table 6.

**Table 6:** Effect of additives on lignin extraction performance

Raw material	DES	Additives	Temperature, time	Lignin extraction performance	Effect	References
Poplar	ChCl: LA	–	120 °C, 12 h	80.4% yield, 94.18% purity	Strong acids provide abundant active protons, promoting ether bond (β-O-4) cleavage and accelerating carbohydrate degradation, thereby enhancing lignin release efficiency	Wu et al. (2021)
		H_3_PW_12_O_40_	120 °C, 4 h	82.2% yield, 89.5% purity		
		FeBr_3_	120 °C, 8 h	80.9% yield, 90.78% purity		
Corncob	ChCl: LA	–	120 °C, 15 h	71.65% yield, 85.62% purity	Catalysis promotes lignin depolymerization and enhances selective separation capability	Yuan et al. (2021)
		Fe_3_O_4_-SiO_2_-V_3_		98.13% yield, 97.09% purity		
Moso bamboo	ChCl: Gly	–	120 °C, 3 h	5.76% delignification ratio	Lewis acids facilitate β-O-4 bond cleavage, thereby enhancing lignin depolymerization	He et al. (2024b)
		FeCl_3_		~ 86% delignification ratio		
		AlCl_3_		~ 83% delignification ratio		

ChCl: choline chloride; LA: lactic acid; Gly: glycerol

The introduction of acidic additives can significantly enhance the catalytic cleavage of LCC linkages and β-O-4 bonds in lignin, thereby reducing the energy barrier required for chemical bond disruption. Previous studies demonstrated that the incorporation of phosphotungstic acid (H_3_PW_12_O_40_) and FeBr_3_ into the ChCl-LA system for poplar pretreatment enabled lignin recoveries of 80.9–82.2% within only 4–8 h, outperforming the 80.4% recovery achieved by the additive-free DES system after 12 h of treatment (Wu et al. 2021). Similarly, a Fe_3_O_4_-SiO_2_-V_3_ composite catalyst was introduced into a ChCl-LA system for corncob pretreatment, significantly improving extraction performance while reducing the required processing severity. Specifically, the extraction temperature was decreased from 150 to 120 °C, and the reaction time was shortened from 18 to 15 h, achieving a maximum lignin recovery of 98.13% (Yuan et al. 2021; Zhang et al. 2022).

The inclusion of additives accelerates extraction kinetics while exerting a profound influence on the microstructure and chemical reactivity of the isolated lignin. An appropriate dosage of Lewis acid can modulate reaction pathways alongside bond cleavage. For instance, introducing optimal amounts of FeCl3 strengthens hydrogen-bonding interactions between DES and lignin, inhibiting non-specific condensation while maintaining efficient depolymerization (Zhao et al. 2025). This selective bond cleavage and condensation-suppression mechanism yields lignin characterized by high purity, low molecular weight, and a relatively intact structural framework. Retaining key reactive functional groups while exhibiting low polydispersity, this tailored lignin serves as an ideal high-reactivity precursor for synthesizing high-performance phenolic resin adhesives, antioxidants, and polyurethane materials.

Although chemical additives can significantly improve lignin extraction efficiency, their application is still associated with several practical limitations. While high concentrations of additives or strongly acidic agents can accelerate the cleavage of lignin bonds, they may also promote undesirable secondary condensation reactions of dissolved lignin-derived intermediates, leading to excessive carbonization, reduced lignin solubility, and decreased molecular reactivity (Shen et al. 2022). From the perspectives of solvent recovery and industrial scale-up, the introduction of additives considerably increases the complexity of DES regeneration and purification after lignin precipitation (del Mar Contreras-Gámez et al. 2023). Meanwhile, residual metal ions introduced into the system may contaminate the recovered lignin, thereby limiting its applications in high-value fields such as advanced functional materials and high-purity products. Furthermore, increased system acidity can substantially aggravate equipment corrosion, resulting in higher requirements for corrosion-resistant materials and increased maintenance costs. Therefore, the catalytic benefits provided by chemical additives must be carefully balanced against the additional costs associated with solvent recovery, purification, and equipment maintenance during process design.

### Other auxiliary methods

Although chemical additives can shorten extraction times and lower reaction temperatures, their overall efficacy and downstream solvent recovery present clear limitations. Therefore, physical-assisted technologies, such as microwave and ultrasound irradiation, have recently been explored as effective approaches to improve the efficiency of DES-based lignin extraction. To further elucidate the influence of these auxiliary technologies on lignin extraction performance, the effects of microwave- and ultrasound-assisted processes on lignin recovery were summarized in Table 7.

**Table 7:** Effect of other auxiliary methods on lignin extraction performance

Raw material	DES	Assisted method	Processing conditions	Lignin extraction performance	Effect	References
Corn stover	ChCl: LA	–	120 °C, 6 h	51.84% yield, 89.15% purity	Microwave irradiation induces rapid vibration of polar molecules, accelerating heat transfer and LCC linkage cleavage, thereby drastically shortening reaction time	Liu et al. (2020b)
		Microwave-assisted	800 W, 45 s	80% removal rate		Chen and Wan (2018)
Larch	ChCl: Acetic acid	–	130 °C, 3 h	93% yield	Microwave radiation enhances the polarity and ionic mobility of DES molecules, intensifying the mass transfer process. Compared to conventional heating, reaction time is reduced by 87% and energy consumption is lowered by 93.5%	Meindl et al. (2022)
		Microwave-assisted	30 min	96% yield		
Cotton straw	ChCl: FA	Ultrasound-assisted	32 kHz, 30 min, followed by oil bath heating at 180 °C for 3 h	87.7% yield, 97.3% purity	Ultrasonic cavitation disrupts the cell wall architecture, increasing the contact area between the solvent and biomass. Compared to native lignin, the extracted lignin exhibits a lower molecular weight and a more uniform molecular weight distribution	Xu et al. (2023)
Barley straw	ChCl: LA	Ultrasound-assisted	25 kHz, 80 °C, 5 h	61.59% solubilisation	Increasing the ultrasonic frequency (from 25 to 45 kHz) can further enhance the lignin removal efficiency	Pradhan et al. (2025)
			45 kHz, 80 °C, 5 h	70.54% solubilisation		

ChCl: choline chloride; LA: lactic acid; FA: formic acid

The advantage of microwave heating lies in its ability to apply an electromagnetic field, which facilitates the vibration and rotation of polar molecules within lignocellulosic biomass. This accelerates heat release while preventing heat loss and ensuring uniform heating (Mankar et al. 2021, 2022). Furthermore, it also enhances the molecular polarity of DES and maximizes its ionic properties, leading to improved lignin extraction efficiency within shorter reaction times (Liu et al. 2017). For example, introducing microwave assistance into ChCl-formic acid and ChCl-LA systems achieved delignification efficiencies of 80–90.1% within only several minutes or even seconds, while producing lignin fractions with purities exceeding 95% (Ceaser et al. 2023; Chen and Wan 2018; Mankar et al. 2022). Compared with conventional conductive heating, microwave-assisted extraction can reduce processing time by more than 80%, resulting in significant reductions in energy consumption (Meindl et al. 2022).

Ultrasound-assisted extraction primarily relies on acoustic cavitation and high-frequency mechanical oscillation to disrupt the surface structure of lignocellulosic biomass and enlarge the interfacial area between DES and biomass substrates, thereby facilitating lignin transfer into the solvent phase (Gavrila et al. 2024). Previous studies have demonstrated that ultrasound-assisted extraction (e.g., 32 kHz for 30 min) of cotton stalks, wheat straw, and barley straw achieved lignin recoveries of up to 87.7% or even near-complete dissolution, while maintaining controlled molecular weights and narrow molecular weight distributions of the extracted lignin (Malaeke et al. 2018; Pradhan et al. 2025; Xu et al. 2023). Furthermore, the combined application of microwave and ultrasound irradiation for the pretreatment of garlic peel and scallion root wastes exhibited significant synergistic effects, achieving over 90% lignin removal while effectively preserving cellulose fractions (Ji et al. 2020). Overall, these physical-assisted technologies share common advantages, including enhanced mass transfer, accelerated energy delivery, and disruption of lignin macromolecular networks. Consequently, lignin fractions with controlled molecular structures, narrow molecular weight distributions, and high chemical reactivity can be obtained, making them promising precursors for the synthesis of high-value-added bio-based materials.

Although physical-assisted enhancement methods such as microwave and ultrasound demonstrate significant advantages at the laboratory scale, they still face substantial engineering challenges and technical constraints during scale-up toward industrial applications. In microwave-assisted processes, microwave irradiation exhibits limited penetration depth (typically 2–10 cm) in highly viscous DES and high-solid-loading biomass matrices, which may result in non-uniform temperature distribution in large-scale reactors. Such uneven heating can induce localized overheating, causing excessive lignin carbonization and degradation of DES components (Fernández 2011; Klinger et al. 2018; Menéndez et al. 2010; Sun et al. 2016). For ultrasound-assisted processes, acoustic energy attenuation with increasing propagation distance makes it difficult to establish uniform and stable cavitation fields in large-volume reactors, thereby restricting reactor capacity and scale-up potential (Kälkäjä et al. 2025). Therefore, although physical-assisted strategies offer high efficiency and effective structural modification capabilities at the laboratory scale, their practical implementation requires a comprehensive balance among extraction performance, energy efficiency, equipment design, and industrial scalability (Table 8).

**Table 8:** Conditions and effectiveness of DES for lignin extraction from agricultural and forestry waste

Raw material	DES	Additive	Assistance method	Molar ratio	Temperature (°C)	Time (h)	Solid–liquid ratio	Lignin yield	Purity of lignin	References
Wheat straw	ChCl: LA			1:2	150	6	1:20	81.54%	91.33%	Ma et al. (2022)
	Pyrazole: LA			1:1	120	9	1:10	80%	97.20%	Lin et al. (2023)
	K_2_CO_3_: Gly	HCl		1:5	100	16	1:30		90.69%	Yue et al. (2022)
Cotton straw	ChCl: FA		Ultrasound	2:2	180	3	1:10	87.70%	93.70%	Xu et al. (2023)
	ChCl: FA: AlCl_3_			1:1:0.05	100	6	1:10	95.60%	95%	Li et al. (2025c)
Poplar	ChCl: Gly	Water, H_2_SO_4_		1:2	140	2–4	1:10	47.50%		Zhang et al. (2024)
	Choline Lactate: LA			1:10	140	12	1:30	90.13%	97.10%	Liu et al. (2020a)
	Zncl_2_: LA			1:10	120	3	1:20		92.10%	Bai et al. (2022)
	ChCl: Benzoic acid			1:0.8	140	3	1:14	46.40%	87.60%	Li et al. (2024)
	ChCl: LA	Phosphotungstic acid		1:10	120	12	1:20	82.20%	89.50%	Wu et al. (2021)
	ChCl: LA	FeBr_3_		1:10	120	12	1:20	80.90%	90.78%	Wu et al. (2021)
	ChCl: OA		Microwave irradiation	1:1	80	2 min	1:20	78.20%	96.30%	Liu et al. (2017)
	Pyridine Hydrochloride: EG: AlCl_3_			1:4:0.2	70	6	1:15	36.80%		Wang et al. (2025b)
Bagasse	ChCl: LA			1:10	120	6	1:20	57.82%	93.73%	Wang et al. (2024)
	ChCl: OA			1:1	109	41 min	1:20	35.33%		Sunar et al. (2024)
	ChCl: Trifluoroacetic acid			1:1.5	111	43 min	1:20	39.50%		Sunar et al. (2024)
Rice straw	ChCl: Tartaric acid			1:4	117	137 min	1:10	45%		Sunar et al. (2025)
	ChCl: OA: LA		Microwave irradiation	1:0.5:1		4 min	1:30		86.80%	Yan et al. (2023)
Corn straw	ChCl: LA			1:2	120	6	1:20	51.84%	89.15%	Liu et al. (2020b)
	ChCl: FA: Maleic acid			1:1.5:0.5	130	3	1:10	76.30%	93.50%	Lu et al. (2022a)
	ChCl: Urea: Ethylenediamine	Water		1:1.4:0.6	110	1.5	1:15		91.53%	Ma et al. (2025a)

ChCl: choline chloride; LA: lactic acid; Gly: glycerol; FA: formic acid; OA: oxalic acid; EG: ethylene glycol

## Effects of DES treatment on the structural characteristics changes of lignin

The impact of DES treatment on the chemical structure of lignin is reflected not only in substantial changes in the distribution and abundance of functional groups but, more importantly, in the selective cleavage and modification of key linkages within the lignin macromolecular backbone, particularly ether linkages and C–C bonds (See Table 13). Since the composition of DES and processing conditions determine the interactions between DES and lignin, different DES systems can promote lignin depolymerization, preserve lignin structures, or trigger condensation reactions to varying extents. These molecular-level structural transformations further influence the functional group distribution, molecular weight, thermal stability, and chemical reactivity of lignin, ultimately determining its potential applications in polymeric materials, functional additives, and fine chemicals.

### Changes in functional groups

In DES-based lignin extraction, changes in lignin functional groups serve as key indicators of structural modification. Compared with conventional industrial lignin, DES-extracted lignin generally exhibits a richer and more chemically accessible functional group composition, including phenolic hydroxyl groups, aliphatic hydroxyl groups, and methoxy groups. These functional groups strongly influence the chemical reactivity and subsequent application potential of lignin (Chio et al. 2019).

#### Phenolic hydroxyl groups

Phenolic hydroxyl is the most reactive functional group in lignin, mainly residing on the aromatic rings of its structural units (Ma et al. 2020). Owing to its robust hydrogen-donating ability and high reactivity, phenolic hydroxyl rich lignin displays enhanced antioxidant performance and UV absorption, making it particularly advantageous for applications in antioxidant materials, UV blockers, and substitutes for phenol–formaldehyde resins (He et al. 2025). Table 9 summarizes the variations in the phenolic hydroxyl content of lignin extracted using DES under different reaction conditions.

**Table 9:** Phenolic hydroxyl content under different reaction conditions

Raw material	DES	Temperature (°C)	Time (h)	Phenolic hydroxyl content (mmol/g)	References
Willow	ChCl: LA (1: 10)	120	6	1.96	Lyu et al. (2018)
			12	2.05	
			18	1.68	
			24	1.59	
Oil palm empty fruit bunch	ChCl: LA (1: 1)	120	8	3.72	Tan et al. (2019)
	ChCl: LA (1: 10)			3.58	
	ChCl: FA (1: 2)			2.66	
Oil palm fronds	ChCl: Gly (1: 3)	150	6	0.738	Amran et al. (2025)
	ChCl: Gly (1: 5)			1.426	
Empty fruit bunches	ChCl: Gly (1: 3)			0.639	

ChCl: choline chloride; LA: lactic acid; Gly: glycerol

DES systems can regulate the phenolic hydroxyl content of lignin by controlling the cleavage of key chemical linkages within the lignin structure (Shen et al. 2020). Under relatively mild conditions, DES treatment can preserve the native lignin architecture to a greater extent, facilitating the production of structurally intact polymeric precursors. For example, pretreatment of poplar wood using a pyridine hydrochloride–EG–AlCl_3_ DES system (70 °C, 6 h) achieved a delignification rate of 70.4%, while the recovered lignin retained native structural features, including well-preserved β-O-4 linkages (42.1 per 100 aromatic units) and a high hydroxyl content (4.97 mmol/g) (Wang et al. 2025b). In contrast, stronger acidic conditions or higher temperatures promote ether bond cleavage, increasing the generation of free phenolic hydroxyl groups and thereby enhancing lignin reactivity (Arshad et al. 2025). Wang et al. (2024) demonstrated that the total phenolic hydroxyl content of eucalyptus lignin obtained by choline chloride–lactic acid (ChCl-LA) DES pretreatment increased from 0.82 mmol/g in enzymatically treated lignin to 2.07 mmol/g, which was mainly attributed to DES-induced β-O-4 bond cleavage and the formation of terminal phenolic hydroxyl structures. Furthermore, after treatment at 120 °C for 4 h, sugarcane bagasse lignin extracted using ChCl-FA-OA exhibited a 35.4% higher phenolic hydroxyl content than that obtained using ChCl-FA-EG, reaching 2.42 mmol/g (Cao et al. 2024).

However, excessively acidic environments may induce lignin condensation reactions, leading to a decrease in the available phenolic hydroxyl groups. Tan et al. (2019) demonstrated that during the extraction of lignin from oil palm empty fruit bunches, increasing the ChCl–LA molar ratio from 1:1 to 1:15 enhanced system acidity and promoted lignin condensation, ultimately resulting in a progressive decline in phenolic–OH content. This indicates that precise control over the phenolic–OH content and structural characteristics of lignin can be achieved by tuning DES acidity, hydrogen bond donor (HBD) ratios, and processing severity.

Therefore, variations in phenolic hydroxyl content during DES extraction arise from the combined effects of β-O-4 bond cleavage, structural preservation, and condensation reactions. Through rational DES design, lignin fractions with tailored reactivity and structural characteristics can be obtained.

#### Aliphatic hydroxyl groups

Aliphatic hydroxyl groups are primarily located on the propane side chains of lignin structural units and serve as crucial interaction sites with external molecules (Suzuki et al. 2021). Unlike acid or alkali treatments, DES treatment typically leads to a declining trend in the aliphatic hydroxyl content of lignin. Studies have demonstrated that the aliphatic hydroxyl content was 2.61 mmol/g in lignin following NaOH pretreatment, compared to 0.91 mmol/g after DES treatment (Ji et al. 2021). Similarly, the content was 1.71 mmol/g after acidolysis, but dropped to 1.17 mmol/g following DES treatment (Li et al. 2020). This reduction is attributed to β-O-4 bond cleavage during DES pretreatment, dehydration and acylation reactions occurring during condensation, as well as the oxidation of certain aliphatic hydroxyl groups into formyl groups in the presence of air (Hong et al. 2020; Shen et al. 2016). Table 10 summarizes the variations in the aliphatic hydroxyl content of lignin extracted using DES under different reaction conditions.

**Table 10:** Aliphatic hydroxyl content under different reaction conditions

Raw material	DES	Temperature (°C)	Time (h)	Aliphatic hydroxyl content (mmol/g)	References
Willow	ChCl:LA (1: 10)	120	6	1.46	Lyu et al. (2018)
			12	1.32	
			18	0.94	
			24	0.93	
Acacia wood	LA: CA: ChCl (0.6: 0.3: 0.1)	140	2	0.77	Fernandes et al. (2024)

ChCl: choline chloride; LA: lactic acid; CA: citric acid

This evolutionary trend of aliphatic hydroxyl groups directly dictates the application pathways and compatibility scenarios of DES lignin in downstream high-value polymeric materials. A higher aliphatic hydroxyl content can strengthen the hydrogen-bonding interactions between lignin and the polymer matrix, thereby enhancing its reactivity potential in polymeric materials such as polyurethanes and epoxy resins (Hemmilä et al. 2020; Rubens et al. 2022).

#### Methoxyl groups

Methoxy groups (–OCH_3_) are characteristic functional groups attached to the aromatic rings of lignin, and their abundance and distribution serve as important indicators for differentiating the three major lignin structural units: guaiacyl (G), syringyl (S), and p-hydroxyphenyl (H) units (Sun et al. 2024). Among natural biomass resources, hardwood lignin is mainly composed of S and G units, with a relatively high proportion of S units, resulting in a higher intrinsic methoxy content (Ralph et al. 2019). Softwood lignin predominantly consists of G units (approximately 90%), whereas herbaceous lignin contains H, G, and S units with comparatively lower and more variable methoxy contents (Constant et al. 2016). These inherent differences in lignin composition among biomass feedstocks influence the extent of demethoxylation reactions and the structural characteristics of lignin products obtained during DES extraction.

During DES extraction, acidic systems or DES formulations containing Lewis acids can induce lignin demethoxylation, converting methoxy groups into phenolic hydroxyl groups. Studies on bagasse nanolignin modified by the ChCl–ZnCl_2_ DES demonstrated that, compared with native lignin and untreated nanolignin, the DES-treated lignin exhibited a significantly reduced methoxy content and an increased phenolic hydroxyl content (Younesi-Kordkheili and Pizzi 2024). The intensity of this demethoxylation reaction depends heavily on the native S/G/H composition of the biomass feedstock. When wheat straw, which is rich in G and S units, was used as the feedstock, ChCl-ZnCl_2_ DES treatment increased the total phenolic hydroxyl content of the precipitated lignin by approximately 1.9-fold compared to untreated lignin. Conversely, the methoxy content decreased by 1.6- to 2.2-fold, corresponding to a reduced proportion of S-type units, while the β-O-4 linkage content and molecular weight remained largely unchanged (Hong et al. 2016). Furthermore, reaction temperature and duration are key parameters governing the methoxy content. For red pine, which is rich in G units, the methoxy content of lignin extracted using ChCl–LA and BE–LA systems showed a progressive decline with increasing temperature and prolonged time. This phenomenon may stem from secondary condensation reactions occurring among lignin fragments under severe conditions (high temperature and extended duration) (See Table 11) (Park et al. 2022).

**Table 11:** Methoxy content under different reaction conditions

Raw material	DES	Temperature (°C)	Time (h)	Methoxy content (mmol/g)	Reference
Red pine	–	–	–	3.12	Park et al. (2022)
	ChCl: LA (1: 1)	100	24	2.67	
		120	24	2.63	
		130	6	2.59	
			12	2.34	
			24	2.23	
	Betaine: LA (1: 1)	100	24	2.49	
		120	24	2.47	
		130	6	2.27	
			12	2.25	
			24	2.08	

ChCl: choline chloride; LA: lactic acid

The demethoxylation reaction induced by DES not only modifies the intrinsic chemical reactivity of lignin but also alters the distribution of its pyrolysis products. By converting methoxy groups into more reactive phenolic hydroxyl groups, DES treatment can enhance the formation selectivity of low-molecular-weight phenolic compounds, such as phenol, during lignin pyrolysis (Chen et al. 2025; Hong et al. 2016). This structural transformation increases the reactivity of lignin toward formaldehyde addition and condensation reactions, thereby enabling a higher substitution ratio of petroleum-derived phenol in the preparation of phenol–formaldehyde resin adhesives (Laurichesse and Averous 2014; Younesi-Kordkheili and Pizzi 2024). Therefore, in practical process design, DES catalytic severity must be precisely tailored in conjunction with the initial S:G:H ratio of the biomass feedstock to directionally fabricate lignin precursors tailored for high-value applications.

#### Carboxyl groups

In native lignin structures, the abundance of carboxyl groups (–COOH) is generally relatively low. During DES-based extraction, the carboxyl content of lignin can undergo considerable changes, with the extent and direction of these variations depending strongly on DES composition, extraction conditions, and biomass feedstock characteristics. Therefore, different DES systems may result in either enrichment or depletion of carboxyl groups in the recovered lignin (See Table 12).

**Table 12:** Carboxyl content under different reaction conditions

Raw material	DES	Temperature (°C)	Time (h)	Carboxyl content (mmol/g)	References
Onion skin	ChCl: OA (1: 1)	100	6	1.45	Ghosh and Pramanik (2025)
Potato peel				1.59	
Banana peel				1.26	
Pomegranate peel				0.75	
Tea residue				2.3	
Acacia wood	LA: CA: ChCl (0.6: 0.3: 0.1)	140	2	0.66	Fernandes et al. (2024)

ChCl: choline chloride; OA: oxalic acid; LA: lactic acid; CA: citric acid

Using willow as the feedstock, extraction with ChCl–LA at 120 °C over 6–24 h resulted in a sharp drop in lignin carboxyl content from 0.71 mmol/g to 0.1 mmol/g with prolonged reaction time. This indicates that extended exposure to elevated temperatures induces decarboxylation, causing carboxyl depletion (Lyu et al. 2018). Similarly, investigation into acacia wood showed that lignin extracted via a ternary ChCl–LA–citric acid DES at 140 °C for 2 h yielded a carboxyl content of 0.66 mmol/g alongside an aliphatic hydroxyl content of 0.77 mmol/g (Fernandes et al. 2024). These functional group dynamics are presumably driven by the cleavage of ether linkages (e.g., β-O-4 bonds) within lignin, which generates acidic species during pretreatment (Wang et al. 2020c).

Elevated carboxyl levels act as key reactive sites to engage in physical and chemical crosslinking with multivalent metal ions or polymer backbones, enabling the development of superabsorbent and highly stretchable bio-based hydrogels. Furthermore, this confers superior chelating ability and anionic affinity to lignin, opening broad avenues for its use in advanced dye dispersants, industrial flocculants, and heavy metal remediation materials (Hachimi Alaoui et al. 2023; Hopa et al. 2022; Kazzaz et al. 2019; Qulatein et al. 2024). Therefore, through rational design of DES composition and optimization of extraction parameters, the selective regulation of specific functional groups can be achieved, enabling the preparation of lignin products with tailored structures and functionalities for targeted applications.

### Changes in the characteristic bonds

The various ether bonds and carbon–carbon bonds linking the phenylpropane structural units in lignin form the backbone of its macromolecular three-dimensional network structure, while also determining lignin's integrity and chemical stability (Palumbo et al. 2024). The core chemical mechanism of DES extracted lignin is precisely the selective breaking or modification of these characteristic bonds (Jančíková and Jablonský, 2024). Unlike traditional severe treatments involving strong acids, strong bases, or high temperatures and pressures, DES processing achieves relatively mild yet efficient disassembly through its unique hydrogen-bond network, moderate acidity/alkalinity, and tunable catalytic activity. This yields products with distinct structural characteristics compared to industrial lignin.

#### Ether bonds

Ether bonds, particularly β-O-4 bonds, are the most abundant (accounting for approximately 50–70% of connecting bonds) and most easily cleaved linkages in lignin (Wang et al. 2020a). Studies found that after treating poplar with DES containing choline lactate-LA, the β-O-4 ether bonds (Aα and Aγ) remained intact, but the β-O-4 ether bonds between guaiacyl and syringyl structural units were cleaved. This indicates that DES treatment promotes the cleavage of β-O-4 bonds within the lignin macromolecule, thereby enhancing lignin extraction (Liu et al. 2020a). Furthermore, DES systems containing Lewis acidic metal salts like FeCl_3_ can efficiently cleave β-O-4 bonds under relatively mild conditions, enabling selective dissolution and oligomerization of lignin (Li et al. 2026a). The cleavage of β-O-4 ether bonds significantly reduces the molecular weight of lignin and decreases its polydispersity, forming oligomeric lignin. This structure is more soluble, facilitating subsequent chemical conversion and materialization (Lourenço et al. 2025).

#### Carbon–carbon bonds

**Fig. 1:**
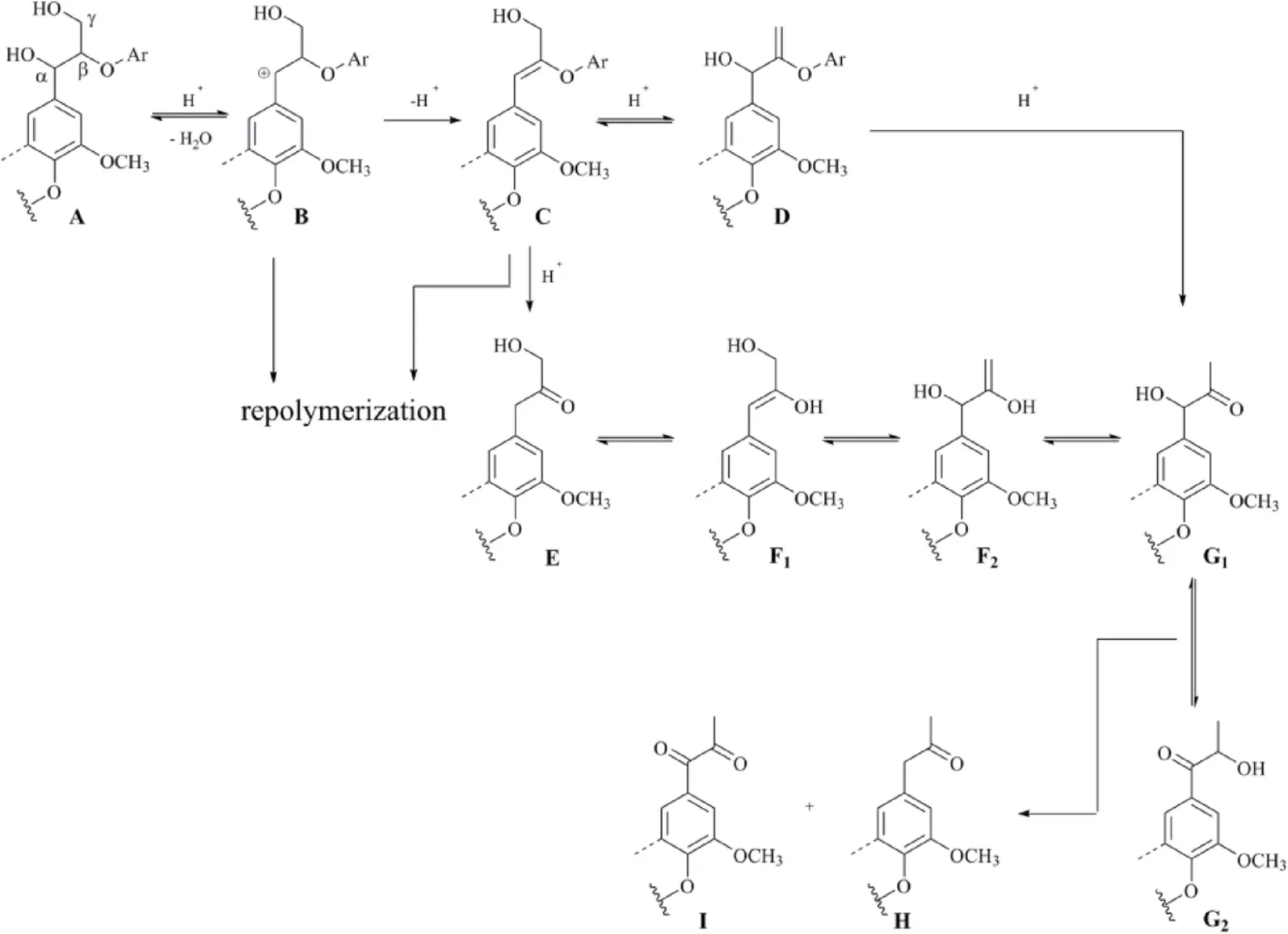
Structural variations of DES-treated lignin. Reproduced from reference (Wang et al. 2020b) (“Unraveling the Structural Transformation of Wood Lignin During Deep Eutectic Solvent Treatment” by Shuizhong Wang is licensed under CC BY 4.0)

Compared to ether bonds, carbon–carbon (C–C) bonds in lignin, such as β–β, β–5, and 5–5 bonds, typically possess higher bond energies and greater stability, making them less susceptible to degradation by conventional acid or base solvents (Questell-Santiago et al. 2020). Studies have shown that after two rounds of DES pretreatment, most C–C bonds are retained and serve as the primary inter-unit bonds in lignin samples, while ether bonds are significantly reduced (Liu et al. 2020a). Cheng et al. (2019) investigated the non-catalytic lignin depolymerization reaction of organic solvent lignin in ethanol-isopropanol mixtures at 270 °C, observing rapid cleavage of α-O-4 and β-O-4 bonds post-reaction while β-β and β-5 bonds remained intact. However, in carboxylic acid-based DES systems combined with elevated temperatures, partial carbon–carbon bond cleavage may also occur. Chen et al. (2019a) observed that a DES composed of ChCl-LA (1:9) not only cleaved ether bonds in poplar lignin but also decomposed some C–C bonds (β-β and β-5) within the lignin. DES composed of ChCl with LA, OA, or malonic acid demonstrated the potential to break carbon–carbon bonds in addition to β-O-4 bond cleavage, further enhancing lignin separation (Yu et al. 2022a) (Fig. 1) (Table 13).

**Table 13:** Effect of DES treatment on the chemical structure of lignin from different raw materials

Raw material	DES	Temperature (°C)	Time (h)	Change of functional group	Change of characteristic key	References
Willow	ChCl:LA (1:10)	120	12	Phenolic hydroxyl structure enrichment	Part of the β-O-4 bonds were cleaved	Li et al. (2017)
	ChCl:LA (1:10)	120	6, 9, 12, 18, 24	The total phenolic hydroxyl content reached its peak at 12 h; as extraction time increased, the carboxyl content decreased	β-O-4 bond was eliminated	Lyu et al. (2018)
Bamboo	ChCl: p-toluenesulfonic acid (1:1)	120	3	There are abundant hydroxyl, carboxyl and carbonyl groups	The β-O-4 bond is severely damaged, C–O and C–C bonds are broken	Song et al. (2024)
Bagasse	ChCl: OA: Propylene glycol (1:1:1)	90	4	The carboxyl group reacts with the hydroxyl group in cellulose to form monoesters or crosslinked diesters	β-O-4 bond is broken, β-β and β-5 bonds are in trace amounts	Yang et al. (2022)
Corn straw	ChCl: OA: Maleic acid (1:1.5:0.5)	90, 100, 110, 120, 130	1, 1.5, 2, 2.5, 3	The phenolic hydroxyl group was well preserved, and the methoxy group was not severely damaged	The β-O-4, β-β and β-5 bonds exist	Lu et al. (2022a)
Wheat straw	ChCl:ZnCl_2_(1:2)	80	1	In precipitation fractionation, the total content of phenolic hydroxyl groups increases, while that of methoxy groups decreases	The β-O-4 bond remains unchanged	Hong et al. (2016)

ChCl: choline chloride; LA: lactic acid; OA: oxalic acid; CA: citric acid

## Effects of DES treatment on changes in the physicochemical properties of lignin

**Fig. 2:**
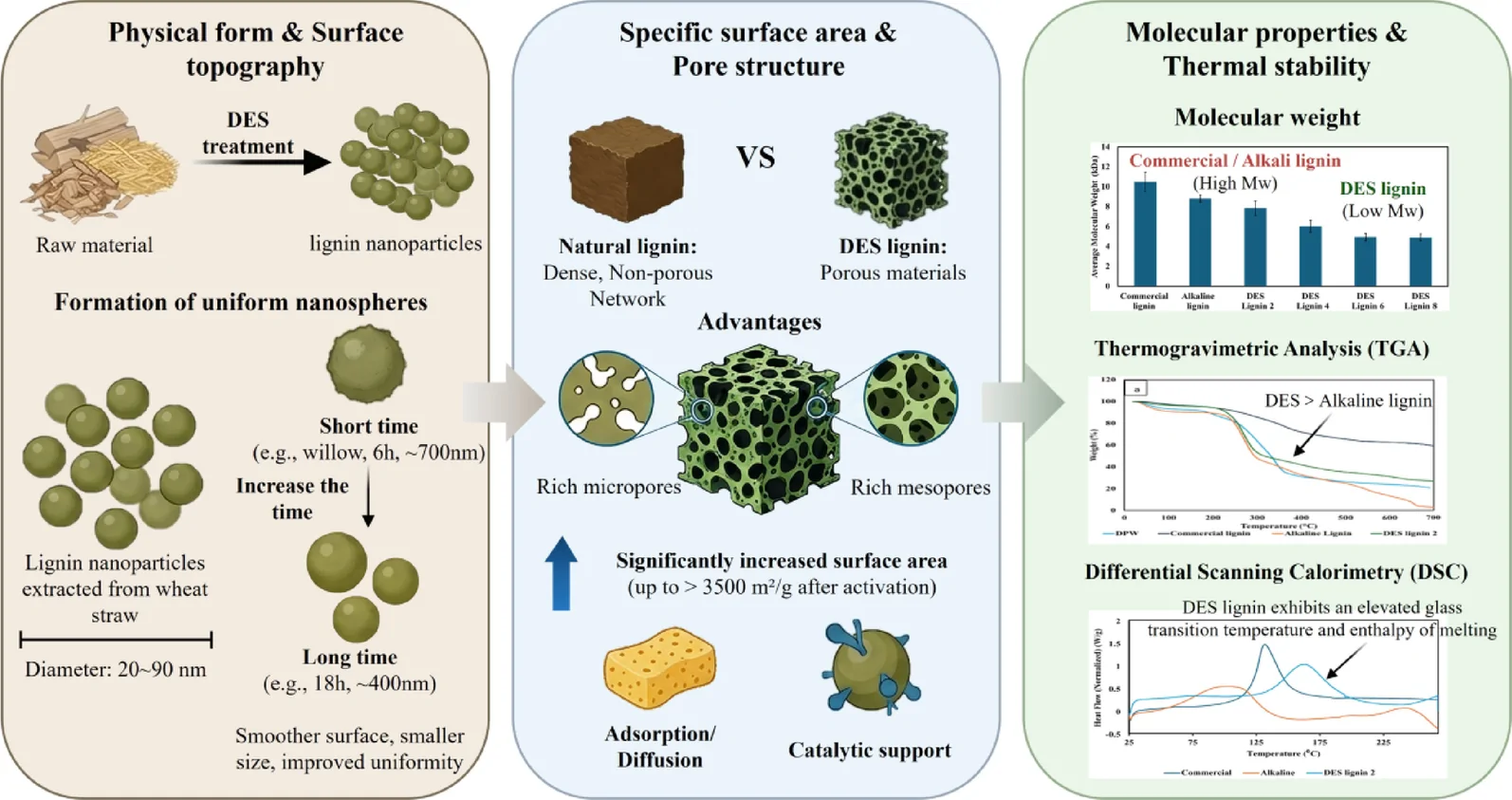
Effects of DES treatment on the physicochemical properties of lignin. Molecular properties & Thermal stability reproduced from reference (Dandash et al. 2025) (“Lignin Extraction from Date-Palm Waste for Structural and Thermal Applications: A Comparative Study of Alkaline and Deep Eutectic Solvent Pretreatments” by Alaa A. Dandash, used under CC BY 4.0/Cropped from original)

Given the wide distribution, diverse origins, and complex compositions of natural lignocellulosic biomass, lignin fractions obtained through different extraction strategies exhibit significant differences in physical morphology, surface characteristics, specific surface area, pore architecture, molecular structural features, and thermal stability, which consequently endow them with distinct potentials for downstream high-value applications (see Fig. 2).

### Changes in physical form and surface morphology

Lignin extracted using DES typically forms spherical or near-spherical particles, with sizes generally ranging from tens to hundreds of nanometers and exhibiting relatively uniform distribution. Studies have revealed that lignin obtained from bamboo, wheat straw, and poplar exhibits a nanostructure, with particles presenting regular spherical shapes (Zhang et al. 2023). Studies on willow lignin extracted using ChCl-LA (1:10) DES revealed that as extraction time increased from 6 to 18 h, the particle size of the isolated lignin decreased from approximately 700 nm to about 400 nm. indicating that extended DES treatment promotes finer, more uniform lignin particles. Concurrently, the particles exhibited relatively smooth surface morphologies, suggesting possible self-assembly of lignin molecules during precipitation from solution to form stable nanoparticle structures (Lyu et al. 2018). Lignin extracted from wheat straw comprised well-dispersed nanoparticles with a size distribution ranging from 70 to 90 nm (Lou et al. 2019). In DES systems containing FeCl_3_, the resulting lignin particles exhibited a narrower size distribution of 21–76 nm (Luo et al. 2025).

It can be seen, DES extraction of lignin is suitable for producing lignin nanoparticles, which holds significant importance for its application in biorefining.

### Changes in specific surface area and pore structure

It is well known that the specific surface area and pore structure of materials directly influence their functionality and applicability (Ahmad et al. 2014). However, cellulose, hemicellulose, and lignin in the plant cell wall interact closely to form a dense network, resulting in low specific surface area and undeveloped pore structure of native lignin, which severely limits its high-value utilization. During DES extraction of lignin, solvent interactions promote chain scission, molecular weight reduction, and self-assembly or re-precipitation of lignin, thereby altering its pore structure and specific surface area (Xie et al. 2025). Although reports on directly measuring the specific surface area of lignin are still limited, relevant studies have demonstrated that lignin derivatives obtained through DES extraction, or porous materials prepared using them as precursors, exhibit high specific surface areas. For example, lignin extracted from wheat straw in ChCl-LA at 120 °C for 4 h had a specific surface area of 8.8 m^2^/g. and KOH-activated treatment yielded a multi-porous material with a specific surface area as high as 3577.3 m^2^/g and a pore volume exceeding 1.5 cm^3^/g, featuring abundant micropores and mesopores. This demonstrates the potential of DES-extracted lignin to form hierarchical pore structures (Xu et al. 2024). Lignin treated with DES composed of toluene sulfonic acid and ChCl yielded a material with a specific surface area of 1026 m^2^/g when used as a carbon material precursor, significantly higher than that obtained without DES treatment (Li et al. 2023a). Lignin separated from DES and crosslinked with formaldehyde into a porous gel exhibits a significantly enhanced specific surface area, reaching nearly 173 m^2^/g in a porous structure. This high porosity facilitates molecular diffusion and enhances adsorption capacity (Lin 2020).

Overall, DES-extracted lignin exhibits a significantly increased specific surface area and more developed pore structure, enhancing its functionality in high-value applications such as adsorption, catalysis, composite materials, and energy storage.

### Changes in molecular properties and thermal stability

DES extraction typically significantly reduces the molecular weight of lignin, transforming it from a complex macromolecular network into smaller, more uniformly distributed fragments. Studies using date palm waste as feedstock have shown that, the weight-average molecular weight (Mw) of commercial lignin can reach as high as 10,490 g/mol, whereas after DES extraction, the Mw drops to below 5,000 g/mol, and the polydispersity index (PDI) decreases significantly (PDI < 2) (Dandash et al. 2025). In willow wood, treatment with ChCl-LA (1:10) DES at 120 °C for 6–24 h reduced the number-average molecular weight (Mn) of lignin from 1348 g/mol to 920 g/mol and the Mw from 1806 g/mol to 1042 g/mol. Molecular weight progressively decreased with extended extraction time, while the PDI decreased from 1.34 to 1.13, indicating a progressively narrower and more uniform molecular weight distribution (Lyu et al. 2018). Similar results were confirmed in poplar wood studies, where DES-extracted lignin exhibited lower Mw (1805 g/mol) and Mn (971 g/mol) values, and a narrower PDI distribution (PDI < 2.0) (Liu et al. 2020a).

Furthermore, compared to traditional alkaline and sulfurization methods, DES extraction is more advantageous for obtaining low-molecular-weight, highly uniform lignin. For instance, lignin extracted using a ternary DES system of ChCl-5-sulfonylsalicylic acid-Gamma-Valerolactone exhibits a significantly lower molecular weight than that obtained via the sulfate method, with a smaller PDI of 1.55 (Liu et al. 2025). The Mw of lignin extracted using ChCl-FA DES was 4,900 g/mol, whereas the Mw of lignin treated with NaOH was 8,830 g/mol (Dandash et al. 2025). These studies collectively demonstrate that the DES system effectively promotes the cleavage of key bonds such as β-O-4, yielding lignin fragments with lower molecular weights, narrower molecular weight distributions, and more uniform structures—factors critical for subsequent modification.

Thermal stability is one of the key physical properties for evaluating the structural integrity of lignin and its potential for high-value utilization. During pyrolysis, the thermal decomposition of lignin is typically divided into three stages: Stage I (< 200 °C) primarily involves the evaporation of water and small-molecule impurities; the second stage (200 °C–600 °C) constitutes the primary degradation zone, representing the core phase where various internal bonds within lignin break, accompanied by the formation of phenolic compounds, gases, and other volatile products; the third stage (> 600 °C) is the carbonization process, where primary products transform from volatile substances into biochar (Chen et al. 2020a; Li et al. 2022b; Wang et al. 2024). Research indicates that lignin extracted using DES typically exhibits higher thermal stability compared to lignin obtained through traditional alkali extraction methods. A study on date palm lignin revealed that alkali-extracted lignin reached its maximum decomposition rate at 236 °C, whereas DES-extracted lignin exhibited its peak decomposition rate around 270 °C. This indicates that the DES lignin possesses a more stable aromatic skeleton and is less prone to pyrolysis, thereby exhibiting superior thermal stability. Furthermore, the study observed a slight decrease in the maximum decomposition rate with extended DES pretreatment time and reduced lignin molecular weight, indicating that thermal stability is closely related to lignin molecular weight and degree of fracture (Dandash et al. 2025).

The thermal stability of lignin also varies across different DES systems. Li et al. (2022a) found that, at a endpoint of testing at 0 °C, the weight loss of lignin after degradation with ChCl-p-toluenesulfonic acid was 71.45%, whereas the weight loss rates for lignin treated with ChCl-Gly, ChCl- OA, ChCl-EG, a mixture of ChCl-FA and acetic acid, ChCl-urea, and ChCl-acetic acid ranged from 41.71% to 46.51%. Furthermore, DES extraction not only alters the thermal decomposition temperature characteristics of lignin but also significantly influences its thermal degradation behavior. For instance, lignin nanoparticles (DLNP) obtained through DES depolymerization exhibited markedly enhanced thermal stability compared to conventional alkali lignin (KL), with an initial decomposition temperature elevated by approximately 20 °C and a main decomposition temperature rising from 352.0 °C to about 388.5 °C. They also exhibit higher residual carbon content, which is primarily attributed to the formation of more highly condensed aromatic structures during DES treatment. These structures enhance lignin's resistance to thermal degradation (Kim et al. 2025).

In summary, DES treatment enables simultaneous controllable depolymerization and enhanced thermal stability of lignin, providing a superior structural foundation and thermal processing adaptability for its application in high-temperature fields, such as carbon materials, flame-retardant materials, and structural composites.

## The impact of DES treatment on the high-value utilization of lignin

The composition and processing conditions of DES allow for the targeted regulation of lignin structure and properties. These variations in structure and physicochemical characteristics directly alter lignin's reactivity, processability, and functional properties during subsequent processing, laying the foundation for its high-value utilization. Currently, the primary pathways for lignin valorization include catalytic conversion into phenolic monomers as platform chemicals, fabrication of functional composite materials, and use as carbon precursors for energy storage materials (See Table 14). These application scenarios highlight the immense potential of transforming lignin from a waste stream into high-value-added products.

**Table 14:** Main high-value applications of DES-treated lignin and their technical challenges

High-Value application direction	Typical products/Applications	Advantages of DES-treated Lignin	Major technical challenges	References
Catalytic Depolymerization to Phenolic Monomers	Production of phenolic monomers such as phenol, 4-ethylphenol, and guaiacol, which serve as feedstocks for fine chemicals, bio-oil, etc	Promotes depolymerization of lignin macromolecules, selectively cleaves β-O-4 linkages, regulates pyrolysis product distribution, and increases the exposure of active functional groups, facilitating subsequent catalytic conversion	(1) High temperatures required for many DES extractions tend to disrupt lignin structure, inducing undesirable condensation or degradation (2) Reactive lignin fragments generated during catalytic depolymerization are prone to condensation and repolymerization, forming stable C–C bonds and reducing target phenolic monomer yields (3) Most current studies remain at the laboratory scale. Scaling up DES pretreatment and lignin depolymerization from gram to kilogram or metric-ton levels poses engineering hurdles in mass/heat transfer and reaction control	Das et al. (2018), Kalhor and Ghandi (2021), Wang et al. (2025a), Yan et al. (2024), Yue et al. (2023)
Nanolignin & Polymer Composites	Lignin nanoparticles, polymer composite films, functional hydrogels, phenolic resins, etc	Enhances lignin purity, tunes molecular weight and molecular weight distribution, and increases reactive groups (e.g., hydroxyl and carboxyl groups), thereby improving interfacial bonding with polymer matrices	(1) Precise control over lignin structure and particle size remains difficult, leading to insufficient stability in adsorption performance (2) High DES viscosity and strong interaction with lignin make complete solvent removal challenging by conventional methods, forming amorphous impurity layers on nanomaterial surfaces and degrading purity and surface activity (3) Low-molecular-weight lignin nanoparticles exhibit poor thermal stability, restricting their application scenarios	Luo et al. (2021), Provost et al. (2022), Xiao et al. (2026), Younesi-Kordkheili and Pizzi (2024), Zheng et al. (2025)
Energy Storage & Functional Carbon Materials	Supercapacitor electrode materials, electrolyte materials	Effectively preserves the aromatic framework of lignin, increases purity, and tunes molecular architecture, making it suitable as a carbon precursor	(1) Uneven condensation degrees in DES lignin lead to uncontrollable thermal decomposition during subsequent carbonization, causing inhomogeneous pore nucleation and wide pore-size distributions with fluctuating electrochemical performance (2) High DES viscosity and strong interaction with lignin hinder complete solvent removal, leading to heteroatom doping during high-temperature carbonization and impairing subsequent electrical conductivity	Ghosh et al. (2026), Li et al. (2026b), Wysokowski et al. (2024), Zheng et al. (2026)

### Preparation of phenolic compounds

Phenolic compounds themselves are high-value-added green chemicals, applicable in synthetic materials, fragrances, functional chemicals, pharmaceutical intermediates, and more (Martínková et al. 2023). Lignin extracted via DES, owing to its aromatic skeleton, abundant phenolic hydroxyl groups, and relatively low molecular weight, serves as an excellent feedstock for preparing high-value-added phenolic chemicals (see Fig. 2). Compared to traditional extraction methods, DES extraction can more efficiently cleave bonds such as β-O-4, increasing the number of active ends. This provides a favorable structural foundation for subsequent catalytic cracking to convert into monomeric phenolic compounds (Kalhor and Ghandi 2021).

In recent years, increasing research has focused on utilizing DES pretreatment combined with rapid pyrolysis to enhance the yield of phenolic compounds. For instance, Li et al. (2022b) found that a DES composed of ChCl-EG and zinc chloride-EG significantly increased the yields of H-phenols and C-phenols. When willow lignin treated with ChCl-LA DES was pyrolyzed at 450 °C, the relative total phenolic content reached 95.10% (in terms of peak area percentage) among the volatiles detected by Py-GC/MS, in which phenol and guaiacol accounted for the largest proportions at 20.8% and 24.2% (relative peak area), respectively (Lyu et al. 2019). Lignin extracted with FeCl_3_-EG (1:0.3) DES and subjected to rapid pyrolysis yielded phenolic compounds as the main products, with phenol accounting for up to 40%. With increasing FeCl_3_ concentration, H-type phenols increased while G-type phenols decreased, indicating that Fe^3+^ promoted demethoxylation reactions, favoring the formation of structurally simpler H-type phenolic products (Li et al. 2026a). Lignin extracted using an alkaline DES (K_2_CO_3_-EG) exhibited fewer condensed structures and retained more native bonds, yielding higher aromatic monomer contents during subsequent catalytic oxidation or depolymerization processes. Its monomer content increased by approximately 116% compared to lignin extracted via conventional alkaline methods (He et al. 2024a). Furthermore, ChCl-glyoxal can isolate β-O-4 bonds and low-condensation-degree light-colored lignin, achieving approximately 21 wt% aromatic monomer yield via catalytic hydrogenolysis. Among these, 73.6% exhibited propylphenols structures (Li et al. 2025a).

As can be seen, the production of phenolic chemicals from DES-extracted lignin relies not only on the structural regulation of the extraction process itself but also requires integration with subsequent catalytic systems to achieve higher selectivity and yield.

### Functional materials synthesis

Because DES-extracted lignin possesses a low molecular weight, a more uniform structure, and abundant active sites, it shows significant advantages in constructing various high-performance materials. This provides a crucial foundation for subsequent functional material synthesis and exhibits excellent potential for high-value utilization.

In the field of nanostructured materials, DES-extracted lignin can serve as a precursor for preparing nanomaterials. For instance, a study synthesized core–shell lignin silver nanoparticles (AgLNPs) using DES-extracted lignin, which were then embedded into polyvinyl alcohol (PVA) films to form PVA composite films. The composite material exhibited a tensile strength of 37.9 MPa, a static water contact angle of 104.2°, and improved UV blocking capability and antibacterial efficacy (Xu et al. 2025). Luo et al. (2021) also demonstrated that DES-prepared LNPs endowed PVA films with superior UV resistance, thermal stability, and hydrophobicity. ChCl-LA DES was employed to deconstruct coconut fibers. Using an antisolvent method, uniform and smooth coconut fiber lignin nanoparticles (CFLNPs) with outstanding antioxidant activity were obtained, significantly enhancing the thermal stability, mechanical properties, hydrophobicity, antioxidant activity, and antibacterial activity of the nanocomposite films (Zheng et al. 2025).

In the field of functional composite materials, lignin can synergistically construct performance-enhanced systems with other polymers. Several studies indicate that esterification, etherification, amination, and other modifications of lignin can improve its compatibility with plastics, elastomers, and bio-based polymers, thereby strengthening interface bonding and enhancing performance (Ma et al. 2025b). For instance, after extracting lignin using ChCl-LA (1:1) DES and subsequently esterifying it, the modified lignin, when prepared as a composite film with PLA, exhibited a more uniform morphology and stronger interfacial bonding compared to the unmodified lignin composite film (Park et al. 2021). Pine lignin treated with ChCl-LA (1:7) DES and subsequently esterified with succinic anhydride (SAn) was used as an additive. This not only enhanced the mechanical properties of PLA while maintaining its thermal stability, but also improved its antibacterial efficacy and biocompatibility (Yan et al. 2022).

Furthermore, lignin-based materials offer unique advantages in microstructure-controlled preparation. Combined with DES extraction and subsequent regulation strategies, that can be prepared into lignin-derived materials featuring tunable pore sizes, nanospheric or hierarchical porous structures (Xu et al. 2024). Wang et al. (2025c) employed ChCl–LA DES to dissolve lignin, combined with wet electrospinning and SiO_2_ nanoparticle templating, to synthesize nano-carbon fibers with multi-scale pore structures. Hong et al. (2023) prepared hierarchical porous carbon materials by subjecting DES-pretreated eucalyptus lignin mixtures, without separation or purification, to DES thermal carbonization (DTC) and activation, thereby enhancing the carbonization degree and thermal stability of the material.

In summary, the synthesis and application of functional materials from DES-extracted lignin shows strong development potential in terms of practical performance enhancement and sustainable substitution.

### Energy application

Lignin obtained through DES extraction is also emerging as an ideal precursor for energy materials. During thermochemical conversion processes, this type of lignin exhibits high reactivity and conversion rates. Li et al. (2022b) demonstrated that ChCl-EG DES pretreatment significantly increases bio-oil yield during lignin pyrolysis while reducing char residue formation, beneficially enhancing biomass fuel production and quality. For supercapacitor electrode material preparation, several studies leverage the porous structure and high active sites of DES-extracted lignin in their designs. For instance, K_2_CO_3_-EG DES was used to extract lignin from cottonseed meal, followed by gelation to form a lignin/agar gel composite. Through air-carbonization, a porous carbon material was obtained for supercapacitor electrodes, achieving an energy density of 30.2 Wh kg^−1^ and demonstrating stable operation across − 25 °C to 75 °C with excellent energy storage performance (Ren et al. 2025). Wang et al. (2025c) dissolved lignin using a choline-LA DES and performed wet electrospinning. Utilizing SiO_2_ nanoparticles as templates, they prepared hierarchical porous carbon nanofibers. The obtained carbon material achieved a specific capacitance of 237.1 F/g at a current density of 0.5 A/g, and the capacitance retention rate as high as 97.7% after 15,000 cycles. Ma et al. (2021) developed a hierarchical porous carbon for supercapacitor electrodes by carbonizing lignin extracted with a ternary DES of ChCl-urea-Gly in combination with polyethylene glycol diglycidyl ether (PEGDE). This electrode exhibits high specific capacitance (270 F/g at 1.0 A/g and 224 F/g at 0.5 A/g) and excellent stability (7% capacitance loss after 10,000 cycles at 8 A/g).

As can be seen, DES extraction of lignin provides a foundation for developing green energy materials, offering broad research and industrialization prospects, particularly in supercapacitors, batteries, and related fields (Table 14).

## Challenges and limitations

Although DESs exhibit considerable advantages in lignin extraction from agroforestry waste, including highly tunable physicochemical properties, effective preservation of lignin structures, and relatively low environmental impact, several challenges remain to be addressed before their large-scale industrial implementation. These challenges mainly involve the intrinsic high viscosity of DES systems, difficulties associated with solvent recovery and recycling, and the limited capability for precise control over lignin structural evolution during extraction.

### High viscosity and mass transfer limitations

DES systems generally exhibit relatively high viscosities, reaching hundreds to thousands of mPa s at room temperature. During lignin extraction, the dissolution of lignin, hemicellulose-derived compounds, and other biomass components into DES further increases system viscosity, thereby intensifying mass transfer limitations (Zhang et al. 2024). This inherent characteristic often requires elevated temperatures and prolonged reaction times to achieve sufficient lignin dissolution and separation, consequently increasing energy consumption and reducing process efficiency. Although external field-assisted technologies, such as microwave and ultrasound irradiation, have demonstrated effectiveness in laboratory-scale batch experiments by accelerating mass transfer, shortening processing time, and lowering operating temperatures, their performance is strongly influenced by reactor design and biomass distribution. During scale-up to continuous or large-volume systems, limitations such as insufficient penetration depth, non-uniform energy distribution, and poor process controllability remain major challenges for practical implementation (Kumar et al. 2019; Zhou et al. 2025).

### Challenges in solvent recovery and recycling

Currently, the vast majority of research on DES-based lignin extraction remains confined to laboratory bench-scale batch trials, with a notable absence of systematic pilot-scale validation and demonstration project data. Engineering challenges—such as high solvent consumption, low recovery efficiency, and impurity accumulation—could be significantly amplified during industrial scaling, serving as the core bottleneck hindering commercial implementation (Cassoni et al. 2025; Ullah et al. 2023). At the solvent recycling level, existing DES recovery heavily relies on conventional methods such as reduced-pressure distillation and rotary evaporation. These processes are not only energy-intensive, but also suffer from the continuous accumulation of degradation products derived from hemicellulose and lignin after multiple cycles. Consequently, this leads to increased DES viscosity, attenuated hydrogen-bonding capabilities, and compromised delignification performance (del Mar Contreras-Gámez et al. 2023; Yu et al. 2022b). Existing experimental data indicate that after five recycle loops, the delignification rate of the DES system decreases by 14%, demonstrating insufficient long-term stability in solvent performance (Zhang et al. 2025). Concurrently, distinct variations in stability exist among different types of DESs during recycling, yet systematic studies clarifying the relationships between DES composition, cycle number, and extraction performance remain lacking. From an industrial application perspective, DES recovery must simultaneously balance economic feasibility and environmental impact. Although DESs are theoretically recyclable, their actual recovery workflow typically entails multiple steps, including solvent separation, purification, and regeneration. The associated energy demands and equipment investments may ultimately compromise the inherent advantages of DESs as green solvents.

### Limited precision in structural regulation

The core objective of current research predominantly focuses on maximizing lignin extraction yield and purity, while paying insufficient attention to regulating structural evolution mechanisms during the extraction process. Consequently, issues such as poor structural homogeneity of target products and uncontrollable retention of functional groups remain widespread (Liu et al. 2021). Studies have demonstrated that under high-temperature acidic DES environments, the cleavage of β-O-4 linkages is accelerated, leading to a continuous increase in phenolic hydroxyl content and a reduction in molecular weight. This not only alters the intrinsic chemical reactivity of lignin but also restricts its potential for subsequent high-value applications (Li et al. 2021b; Wang et al. 2020b).

Furthermore, most existing studies rely on general-purpose extraction strategies rather than application-oriented DES design. Different downstream applications, including phenolic monomer production, polymer modification, and carbon material preparation, require lignin with distinct structural characteristics, such as specific molecular weight distributions, functional group compositions, and condensation degrees (Chen et al. 2018; Im et al. 2026; Mikkilä et al. 2020). However, current DES extraction approaches still lack the ability to precisely tailor lignin structures according to end-use requirements. This limitation prevents the full exploitation of DES compositional flexibility and contributes to a mismatch between extracted lignin properties and targeted applications.

## Conclusion and future perspectives

### Conclusion

This paper systematically reviews the research progress of DESs in lignin extraction from agricultural and forestry residues, focusing on four key dimensions: DES system design, extraction process optimization, lignin structural characteristics, and high-value applications. Studies demonstrate that by rationally selecting HBA and HBD components, as well as tuning solvent molar ratios and processing parameters, highly efficient lignin liberation from agricultural and forestry residues can be achieved. Simultaneously, active functional groups and β-O-4 linkages are well preserved, yielding lignin with both high reactivity and structural integrity. At the application level, DES-extracted lignin can be converted into high-value-added phenolic monomers via catalytic depolymerization, or utilized as functional precursors or modifying components for synthesizing diverse high-performance materials (such as carbon materials and polymer composites), demonstrating promising application prospects.

On the whole, DES lignin extraction technology is still in its infancy regarding industrial application. Key bottlenecks remain in mass transfer efficiency, solvent circulation, and tailored structural control, calling for urgent breakthroughs via process integration, engineering demonstration, and application-driven design.

### Future perspectives

Addressing the core limitations of current research, the future development of DES-based lignin extraction technology should place greater emphasis on the synergistic advancement of fundamental theoretical research and engineering applications.

1. Developing novel DES systems that combine high extraction efficiency, low cost, superior stability, and ease of recyclability represents a crucial future research direction. In recent years, emerging solvent systems such as natural deep eutectic solvents (NADESs) and functionalized DESs have garnered increasing attention, demonstrating promising application prospects in enhancing selective lignin solubility, reducing environmental footprints, and improving recycling stability. Future work should further optimize DES formulation design to lower system viscosity and enhance mass transfer efficiency. Concurrently, establishing highly efficient, low-energy recovery and regeneration processes is essential to achieve long-term, stable solvent recycling.
2. The development of DES extraction technology should focus more on the synergy between lignin extraction and its high-value utilization. Driven by specific demands across diverse application fields—such as phenolic chemicals, high-performance resins, carbon materials, bio-based polymers, and aviation fuels—future efforts should precisely tailor the molecular weight, functional group composition, and linkage distribution of lignin. This will facilitate a paradigm shift from 'maximizing extraction efficiency' to 'target-driven production of functionalized lignin,' thereby elevating the high-value utilization level of lignin resources.
3. Future research should also intensify efforts on the engineering scale-up of DES extraction processes, establishing a cohesive technological bridge spanning laboratory bench, pilot-scale, and industrial applications. To address critical engineering bottlenecks under high solid-to-liquid ratios—such as increased system viscosity, restricted heat and mass transfer, and challenges in continuous operation—future work must focus on developing continuous-flow reactors, process-intensified mass transfer equipment, and intelligent process control technologies. Furthermore, techno-economic analysis (TEA) and life cycle assessment (LCA) should be integrated to systematically evaluate the resource consumption, energy efficiency, environmental impacts, and economic feasibility of the entire DES extraction process, thereby validating the industrialization potential and sustainability advantages of DES technology from a holistic perspective.

## Data Availability

Not applicable.
